# Calcium Oscillations in Pancreatic α-cells Rely on Noise and ATP-Driven Changes in Membrane Electrical Activity

**DOI:** 10.3389/fphys.2020.602844

**Published:** 2020-11-17

**Authors:** Virginia González-Vélez, Anthony Piron, Geneviève Dupont

**Affiliations:** ^1^Department Basic Sciences, Universidad Autónoma Metropolitana-Azcapotzalco, CDMX, Mèxico, Mexico; ^2^ULB Center for Diabetes Research, Faculté de Médecine, Université libre de Bruxelles (ULB), Brussels, Belgium; ^3^Interuniversity Institute of Bioinformatics (IB2), Brussels, Belgium; ^4^Unit of Theoretical Chronobiology, Faculté des Sciences, Université libre de Bruxelles (ULB), Brussels, Belgium

**Keywords:** computational model, ATP-sensitive potassium channels, action potential, Langevin equation, stochastic channel, plasma-membrane Ca^2+^ ATPase

## Abstract

In pancreatic α-cells, intracellular Ca^2+^ ([Ca^2+^]_i_) acts as a trigger for secretion of glucagon, a hormone that plays a key role in blood glucose homeostasis. Intracellular Ca^2+^ dynamics in these cells are governed by the electrical activity of voltage-gated ion channels, among which ATP-sensitive K^+^ (K_ATP_) channels play a crucial role. In the majority of α-cells, the global Ca^2+^ response to lowering external glucose occurs in the form of oscillations that are much slower than electrical activity. These Ca^2+^ oscillations are highly variable as far as inter-spike intervals, shapes and amplitudes are concerned. Such observations suggest that Ca^2+^ dynamics in α-cells are much influenced by noise. Actually, each Ca^2+^ increase corresponds to multiple cycles of opening/closing of voltage gated Ca^2+^ channels that abruptly become silent, before the occurrence of another burst of activity a few tens of seconds later. The mechanism responsible for this intermittent activity is currently unknown. In this work, we used computational modeling to investigate the mechanism of cytosolic Ca^2+^ oscillations in α-cells. Given the limited population of K_ATP_ channels in this cell type, we hypothesized that the stochastic activity of these channels could play a key role in the sporadic character of the action potentials. To test this assumption, we extended a previously proposed model of the α-cells electrical activity (Diderichsen and Göpel, 2006) to take Ca^2+^ dynamics into account. Including molecular noise on the basis of a Langevin type description as well as realistic dynamics of opening and closing of K_ATP_ channels, we found that stochasticity at the level of the activity of this channel is on its own not able to produce Ca^2+^ oscillations with a time scale of a few tens of seconds. However, when taking into account the intimate relation between Ca^2+^ and ATP changes together with the intrinsic noise at the level of the K_ATP_ channels, simulations displayed Ca^2+^ oscillations that are compatible with experimental observations. We analyzed the detailed mechanism and used computational simulations to identify the factors that can affect Ca^2+^ oscillations in α-cells.

## Introduction

Pancreatic islets respond to changes in blood glucose levels so that β-cells secrete insulin when blood glucose is elevated and α-cells secrete glucagon when it is low. Glucagon mobilizes glucose from the liver and when normoglycemia is reestablished, glucagon release from α-cells is suppressed. Extrinsic and intrinsic factors are involved in glucagon secretion (Briant et al., 2016; Wendt and Eliasson, 2020). Individuals with diabetes often show an impaired glucagon secretion that contributes to their hyperglycaemia (D’Alessio, 2011; Gilon, 2020). However, the detailed mechanism by which α-cells regulate glucagon secretion is not fully understood (Yu et al., 2019).

Pancreatic α-cells are electrically excitable and stimulation of glucagon secretion is secondary to repetitive action potential (AP) firing. In a low glucose medium, AP’s occur with a frequency of ∼1–3 Hz. Depolarization of the α-cell plasma membrane allows Ca^2+^ to enter through voltage-gated Ca^2+^ channels, which leads to the exocytosis of secretory granules of glucagon. In agreement with this mechanism, electrical stimulation of α-cells leads to an increase in cell membrane capacitance due to granule fusion, a well-known Ca^2+^ dependent process that precedes glucagon release (Barg et al., 2000; Voets, 2000; Göpel et al., 2004; González-Vélez et al., 2012).

Electrical activity in α-cells is thus accompanied by an increase in intracellular Ca^2+^ concentration ([Ca^2+^]_i_), which results from the activation of voltage-gated Ca^2+^ channels. Interestingly, this rise in [Ca^2+^]_i_ occurs in the form of oscillations with an average frequency of ∼0.5 min^–1^, which is much lower than that of the AP’s. Parallel measurements of electrical activity and [Ca^2+^]_i_ revealed that each oscillation corresponds to a burst of AP’s and that the amplitude of the Ca^2+^ increase correlates with the frequency of AP’s (MacDonald et al., 2007; Quoix et al., 2009; Le Marchand and Piston, 2010, 2012; Zhang et al., 2013; Kellard et al., 2020). These bursts of electrical activity are separated by quiescent periods during which [Ca^2+^]_i_ is close to basal level. Ca^2+^ oscillations are observed in most α-cells, in low or high glucose medium, although they are much reduced in both amplitude and frequency in high glucose. A key characteristic of these oscillations is their irregularity. Their shape, frequency and amplitude are extremely variable, not only from one cell to another but also in the course of time for one individual cell (Kellard et al., 2020). In this study, we investigated the mechanism responsible for the intermittency of electrical activity and thus for the existence of slow, irregular Ca^2+^ oscillations.

Plasma membrane ATP-sensitive K^+^ channels (K_ATP_ channels) play a key role in controlling α-cells electrical activity, although the details of this control are still actively debated (Gilon, 2020; Zhang et al., 2020). When the amplitude of this current is relatively limited, voltage-gated Na^+^ and Ca^2+^ channels can indeed initiate an AP. The number of K_ATP_ channels simultaneously active is however surprisingly low, of the order of 10 (Rorsman et al., 2014). With such a low number of channels, it is expected that fluctuations of molecular origin would play a key role in the dynamical evolution of the K_ATP_ current and thus of the whole voltage and Ca^2+^ dynamics (Gonze et al., 2018). The low K_ATP_ channel activity in α-cells also results in a very high input resistance, meaning that small currents as those associated with openings of a few ion channels may have a drastic effect on membrane voltage and electrical activity (Rorsman et al., 2014). In agreement with this reasoning, noise-induced APs have been observed in α-cells and theoretically simulated (Diderichsen and Göpel, 2006). Here, we pushed this observation forward and investigated the possibility that fluctuations related to the small K_ATP_ current might be responsible for the intermittent character of electrical activity and hence for the noisy Ca^2+^ oscillations. This hypothesis, which is ideally investigated by mathematical modeling, holds with the observations that α-cells activity is highly variable even at a given external glucose concentration and that there is no “typical” α-cell signature (Kellard et al., 2020). Importantly, we here focus on the mechanism responsible for the existence of slow, irregular Ca^2+^ oscillations and not on the actively debated mechanism of regulation of glucagon secretion. Regulation of glucagon secretion indeed involves both intrinsic mechanisms and paracrine signals. Here, we only take into account intrinsic processes. Since intermittent electrical activity and slow [Ca^2+^]_i_ oscillations have also been observed in *isolated* α-cells (Salehi et al., 2006; Quoix et al., 2009; Tuduri et al., 2009; Le Marchand and Piston, 2010), we indeed reasoned that paracrine signaling is not essential for their existence although it affects their characteristics (Briant et al., 2018b; Kellard et al., 2020).

Mathematical models have been developed to studyα-cells electrical activity and glucagon secretion. Diderichsen and Göpel (2006) used experimental data from patch clamp experiments onpancreatic α-cells located on the surface of intactmouse islets to develop an accurate model of plasma membraneelectrical activity. This model was extended to include firstCa^2+^ dynamics and secretion (Watts and Sherman, 2014), and laterglucagon-like peptide 1 (GLP-1) and adrenaline effects (Montefusco and Pedersen, 2015) as well as the α-cell heterogeneity by introducing realistic cell-to-cell variations in the values of the parameters (Montefusco et al., 2020). A functional identification of the islet cell types based on their electrophysiological characteristics allowed to improve the agreement between experiments and simulations of these models (Briant et al., 2017). Diderichsen and Göpel’s model was also re-used by Grubelnik et al. (2020) to study the link between the deformities in mitochondrial ultrastructure observed in α-cells of type 2 diabetes mellitus mice and glucagon secretion. On the other hand, Fridlyand and Philipson (2012) adapted a model initially developed to describe β-cells dynamics to propose a detailed description of α-cells electrical activity, Ca^2+^ changes, metabolism as well as paracrine and endocrine regulations. The effect of paracrine signaling (Watts et al., 2016; Briant et al., 2018b) on α-cells electrical activity was also investigated in models of pancreatic islets including β- and δ-cells. None of these studies have addressed the possible impact of the low number of K_ATP_ channels, nor the question of the mechanistic origin of cytosolic Ca^2+^ oscillations in α-cells.

The present study is based on the original Diderichsen and Göpel’s model of α-cells electrical activity. This core model is sequentially extended to take into account Ca^2+^ dynamics and random fluctuations of the K_ATP_ current via the Langevin formalism. We found that stochasticity in this current can indeed induce intermittent electrical activity and Ca^2+^ oscillations, but only for unrealistically small values of the opening and closing rates of this K^+^ channel. This theoretical prediction motivated us to further extend the model to take into account the variations of ATP concentrations that result from the activity of the plasma membrane Ca^2+^ ATPases. The resulting changes in ATP, which have been observed experimentally (Li et al., 2015), indeed slow down the dynamics of the K_ATP_ current and allow for intermittent electrical activity and Ca^2+^ oscillations resembling those observed experimentally. Finally, we used the model to investigate the sensitivity of calcium and electrical activities to key factors such as the number of K_ATP_ channels or the rate of Ca^2+^ pumping.

## Model Description

**FIGURE 1:**
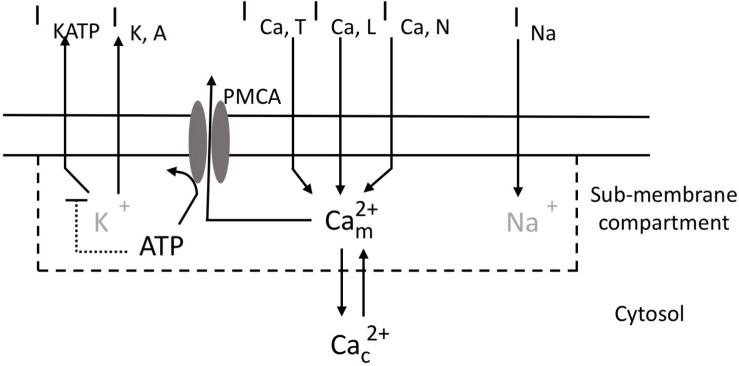
Schematic representation of ionic currents, Ca^2+^ fluxes and ATP consumption in pancreatic α-cells. Transmembrane currents are: I_*KATP*_, the ATP-sensitive K^+^ current; I_*KA*_, the high-voltage activated K^+^ current; I_CaL_, the high-voltage activated L-type Ca^2+^ current; I_CaT_, the low-voltage activated T-type Ca^2+^ current; I_CaN_, the high-voltage activated N-type Ca^2+^ current; I_*Na*_, the voltage-gated Na^+^ current. The model describes the evolution of Ca^2+^ concentration in a fictitious sub-membrane compartment ($C\,a_{m}^{2+}$) and in the cytosol ($C\,a_{\text{c}}^{2+}$). Exchanges between these 2 compartments occur by diffusion. Ca^2+^ is transported from the sub-membrane compartment into the extracellular medium by the ATP-consuming PMCA. ATP increases in the cytosol at a rate that depends on the concentration of extracellular glucose. ATP inhibits the ATP-sensitive K^+^ current (I_*KATP*_). K^+^ and Na^+^ concentrations (in gray) are not explicitly considered in the model.

We base our model (Figure 1) on the mathematical description of the electrical activity of pancreatic α-cells proposed by Diderichsen and Göpel (2006), which was carefully calibrated on experimental data (see also Briant et al., 2017). The original model incorporates an ATP-sensitive K^+^ current (*I*_KATP_) that couples the level of external glucose to the electrical properties of the α-cell plasma membrane, through the sensitivity of the intracellular ATP/ADP ratio to external glucose concentration. It also describes a voltage-gated Na^+^ current (*I*_*Na*_), a delayed rectifying (*I*_KDR_) and a A-type K^+^ current (*I*_KA_), an unspecific leak current (*I*_*leak*_) and a L-type (*I*_CaL_) and a T-type Ca^2+^ current (*I*_CaT_). We also consider an additional type of Ca^2+^ current, because N-type (Quesada et al., 2008; González-Vélez et al., 2010) or P/Q type (Rorsman et al., 2014) Ca^2+^ currents have been shown to play a key role in glucagon secretion in rodents. Here, we arbitrarily chose to incorporate a N-type Ca^2+^ current (*I*_CaN_). Ca^2+^ concentrations are described differently just below the plasma membrane and in the cytoplasm. Ca^2+^ entry via the L-, T- and N-type Ca^2+^ channels increases Ca^2+^ concentration in a hypothetical sub-plasma membrane compartment. From this compartment, Ca^2+^ diffuses into the cytoplasm or leaves the cell via the plasma membrane Ca^2+^ ATPase (PMCA). Ca^2+^ efflux from the cell is thus accompanied by the hydrolysis of ATP into ADP. The resulting decrease in ATP concentration provokes an increase in the K_ATP_ conductance, thereby providing a feedback from Ca^2+^ changes on the electrical properties of the membrane. The main features of the model are described here below. Additional information about the equations and the values of the parameters can be found in the Supplementary Data and in the original study of Diderichsen and Göpel (2006).

### Model of Plasma Membrane Electrical Activity

Electrical activity is described by the following differential equation:

(1) $$\begin{array}{c} \frac{dV}{dt}=-(I_{CaT}+I_{CaL}+I_{CaN}+I_{Na}+I_{KA}+I_{KDR}\\ +I_{KATP}+I_{leak})\text{/}C_{m} \end{array}$$

where *V* is the membrane voltage and *C*_*m*_ is the membrane capacitance set to 5 pF (Diderichsen and Göpel, 2006). For all currents, except for *I*_KATP_ (see section “ATP-Sensitive K^+^ Current and ATP Evolution” below), we use the model proposed by Diderichsen and Göpel (2006) that is based on a Hodgkin-Huxley type description of ion channels and where the activation and inactivation functions have been fitted to experimental data. The high-voltage gated N-type Ca^2+^ current is described as in other studies (Csercsik et al., 2010; González-Vélez et al., 2010).

### Ca^2+^ Dynamics

The evolution of sub-membrane Ca^2+^ concentration, noted *Ca*_*m*_, is modeled as

(2) $$\begin{array}{c} \frac{dCa_{m}}{dt}=f_{r}\cdot \frac{-I_{CaT}-I_{CaL}-I_{CaN}}{2\cdot F\cdot Vol/20}\cdot 10^{6}+\\ V_{b}-V_{p}\frac{Ca_{m}^{2}}{Ca_{m}^{2}+K_{m}^{2}}\cdot f_{1}(ATP)-\text{γ}(Ca_{m}-Ca_{c}) \end{array}$$

where *f*_*r*_ is the fraction of unbuffered Ca^2+^, *F* the Faraday constant and *Vol*, the volume of an α-cell. It is assumed that the sub-membrane shell represents 1/20 of the total volume of the cytoplasm. The factor 10^6^ allows to get concentrations expressed in μM. *V*_*b*_ stands for a basal rate of Ca^2+^ entry, which ensures a ∼0.1 μM Ca^2+^ concentration when voltage-gated Ca^2+^ channels are closed. The third term represents Ca^2+^ pumping out of the cell through PMCA, which can be modeled by a Hill function with *n*_H_ = 2 (Dupont et al., 2016). This active transport is accompanied by ATP hydrolysis. Thus,

(3) $$f_{1}\,\left(A\,T\,P\right)=\frac{A\,T\,P}{A\,T\,P+K_{e}}$$

with *K*_*e*_ being the Michaelis-Menten constant of ATP hydrolysis by the PMCA.

Finally, the last term of Eq. 2 represents Ca^2+^ diffusion into the bulk of the cytoplasm. Given a Ca^2+^ diffusion coefficient of 13 μm^2^/s (Allbritton et al., 1992) and a diameter of 8 μm for an α-cell (Zimny and Blackard, 1975) in which the nucleus occupies ∼70% of the cytoplasm, γ can be estimated to ∼0.01 ms^–1^ (see Supplementary Material).

The evolution of intracellular ATP follows

(4) $$\frac{d\,A\,T\,P}{d\,t}=V_{g\,l\,y}\,\frac{G\,l\,u}{G\,l\,u+K_{g\,l\,u}}-\frac{V_{p}}{2}\,\frac{C\,a_{m}^{2}}{C\,a_{m}^{2}+K_{m}^{2}}\cdot f_{1}\,\left(A\,T\,P\right)-k\cdot A\,T\,P$$

The first term of Eq. 4 is a phenomenological description of glycolysis in the form of Michaelis-Menten rate of ATP production from glucose. The second term represents ATP consumption by plasma membrane PMCA and is thus the same as in Eq. 2 except for the factor 2 that takes into account that one mole of ATP is hydrolyzed for two moles of Ca^2+^ transported. Rate constant *k* describes the consumption of ATP by other intracellular processes.

In agreement with Eq. 1, the evolution of cytoplasmic Ca^2+^ concentration is given by

(5) $$\frac{d\,C\,a_{c}}{d\,t}=\frac{\gamma }{19}\,\left(C\,a_{m}-C\,a_{c}\right)$$

### ATP-Sensitive K^+^ Current and ATP Evolution

In the original model (Diderichsen and Göpel, 2006), the ATP-sensitive K^+^ current is modeled as an ohmic ionic current with a constant conductance, i.e.,

(6) $$I_{K\,A\,T\,P}=g_{K\,A\,T\,P}\,\left(V-V_{K}\right)$$

where *V*_*K*_ is the reversal potential for currents carried by potassium. The channel conductance, *g*_*KATP*_, was considered as a constant parameter and an increase in the extracellular glucose concentration was simulated by a decrease in the value of this parameter.

The main goal of the present study is to assess the effect of noise on the K_ATP_ current that arises in α-cells due to the small number of such channels. *g*_*KATP*_ is thus not considered as a constant parameter anymore. Instead, we stochastically simulate opening and closing of these channels on the basis of the Langevin formalism. These channels can flicker between an open and a closed state, with opening and closing rate constants denoted α and β, respectively. Considering a noise term, the deterministic evolution equation for the fraction of open channels (*s*) becomes:

(7) $$\frac{d\,s}{d\,t}=\alpha \,\left(1-s\right)\cdot f_{2}\,\left(A\,T\,P\right)-\beta \,s+\sqrt{\sigma }\,\xi \,\left(t\right)$$

where *ξ*(*t*) is a random function of time. The last term is a noise function with 0 mean and

(8) $$\sigma =\frac{\alpha \,\left(1-s\right)\cdot f_{2}\,\left(A\,T\,P\right)+\beta \,s}{N_{K\,A\,T\,P}}$$

with *N*_*KATP*_ the number of potentially openable K_ATP_ channels (Fall et al., 2005).

*f*_2_(*ATP*) takes into account that K_ATP_ channels are reversibly inhibited by ATP (Enkvetchakul et al., 2001). We chose the simple expression:

(9) $$f_{2}\,\left(A\,T\,P\right)=\frac{K_{i\,n\,h}}{A\,T\,P+K_{i\,n\,h}}$$

where *K*_*inh*_ is a constant representing the concentration of ATP leading to half-maximal inhibition.

When taking into account the stochasticity of the K_ATP_ channels, the *g*_*KATP*_ conductance appearing in Eq. 6 is now computed as

(10) $$g_{K\,A\,T\,P}=g_{K\,A\,T\,P}^{s}\cdot s\cdot N_{K\,A\,T\,P}$$

with $g_{\text{KATP}}^{\text{s}}$ standing for the unitary conductance of a single ATP-sensitive K^+^ channel.

Together with the equations of the original model (Diderichsen and Göpel, 2006) listed in the Supplementary Data, Eqs 1–10 constitute our computational model that has been integrated in Matlab, using an Euler integration scheme with Δ*t* = 0.012 ms. Bifurcation diagrams have been established using the AUTO package of XPPAUT (Ermentrout, 2002).

### Outline of the Modeling Approach

As explained in detail in the section “Results,” we consider models of increasing complexity to investigate the possible impact of the fluctuations in the K_ATP_ conductance on the existence of Ca^2+^ oscillations in α-cells.

*Model 1* is the model proposed by Diderichsen and Göpel (2006), including N-type Ca^2+^ channels and two additional variables: subplasmalemmal Ca^2+^ (*Ca*_*m*_, Eq. 2 with *f*_1_ = 1) and cytosolic Ca^2+^ (*Ca*_*c*_, Eq. 5). It is thus defined by Eqs 1, 2, and 5.

*Model 2* moreover includes stochasticity in the K_ATP_ conductance through Eqs 7, 8 and 10 that are considered in addition to the equations defining Model 1. *f*_1_ and *f*_2_ are taken equal to 1.

*Model 3* allows to investigate the effect of the Ca^2+^-induced variations in ATP concentration. It is similar to Model 2, except that it includes Eq. 4 to describe the evolution of [ATP]. Changes in the concentration of this nucleotide impact on the evolutions of subplasmalemmal Ca^2+^ via *f*_1_ that is now given by Eq. 3 and of the K_ATP_ channel via *f*_2_ that is now given by Eq. 9.

## Results

### Calcium Changes Induced by Electrical Activity

**FIGURE 2:**
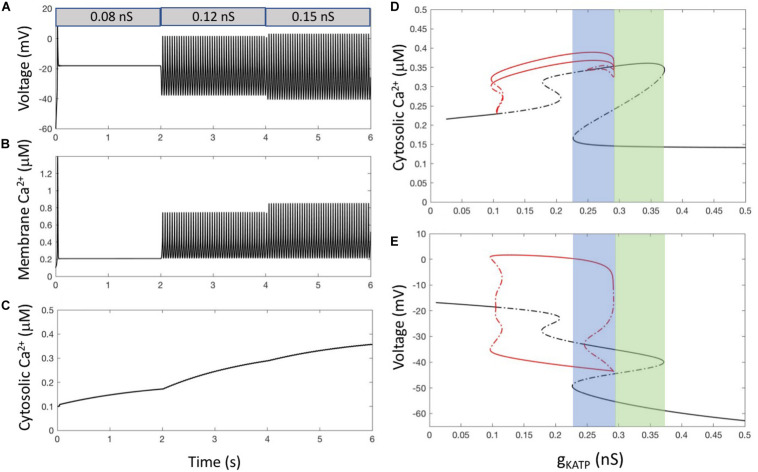
α-cell Ca^2+^ dynamics resulting from electrical activity. **(A)** Membrane voltage for 3 values of *g*_*KATP*_, simulating a decrease in external glucose. From *t* = 0 to 2 s, *g*_*KATP*_ = 0.08 nS and *g*_*leak*_ = 0.2 nS. From *t* = 2 to 4 s, *g_*KATP*_* = 0.12 nS and *g*_*leak*_ = 0.2 nS. For *t* > 4 s, *g*_*KATP*_ = 0.15 nS and *g*_*leak*_ = 0.13 nS. The decrease in *g*_*leak*_ that accompanies the increase in *g*_*KATP*_ allows the model to reproduce the experimentally observed lower maximum voltage reached during repetitive AP’s at high glucose (Montefusco and Pedersen, 2015). **(B)** Corresponding changes in [Ca^2+^] in the sub-plasmalemmal compartment (*Ca*_*m*_). **(C)** Corresponding changes in [Ca^2+^] in the cytosol (*Ca*_*c*_). Because diffusion is much slower than electrical activity, cytosolic concentration does not follow the Ca^2+^ spikes occurring just below the membrane. **(D,E)** Bifurcation diagrams showing the evolution of *Ca*_*c*_ **(D)** and voltage **(E)** when changing *g*_*KATP*_. In both panels, black lines indicate steady states, while red lines show the minimum and maximum values reached during limit cycle oscillations. Plain lines indicate stable solutions and dashed lines instable ones. Bifurcation diagrams have been established using the AUTO package of xppaut (Ermentrout, 2002). For all panels, equations are those corresponding to *Model 1* (see section “Outline of the Modeling Approach”) with the default values of parameters listed in Supplementary Tables 1, 2 of the Supplementary Material, except for the indicated values of *g*_*KATP*_ and *g*_*leak*_. In panels **(D,E)**, *g*_*leak*_ = 0.2 nS.

When α-cells are electrically active, [Ca^2+^]_i_ first rises just beneath the plasma membrane since Ca^2+^ is entering through voltage-gated channels located in this membrane. Sub-plasmalemmal Ca^2+^ is immediately buffered and diffuses in the cytosol where Ca^2+^ concentration is low at rest (∼100 nM). This attenuates Ca^2+^ increases below the membrane and transmits signaling to the rest of the cell. Additionally, sub-membrane Ca^2+^ is pumped out of the cell by Ca^2+^ ATPases. *Model 1* takes these fluxes into account and combines a description of α-cell electrical activity with equations for the evolution of subplasmalemmal (*Ca*_*m*_, Eq. 2) and cytosolic Ca^2+^ (*Ca*_*c*_, Eq. 5). Simulations of *Model 1* show that each AP generates one spike of Ca^2+^ in the subplasmalemmal space (Figures 2A,B). Considering that this fictitious compartment occupies 1/20 of the α-cell volume, corresponding to a shell thickness of ∼100 nm, Ca^2+^ increases up to a level between 0.5 and 1 μM depending on the value of *g*_KATP_. A supra-threshold increase in *g*_KATP_ induces repetitive changes in *Ca*_*m*_, whose frequency and amplitude are fixed by the value of this conductance. Because of the relative slowness of diffusion, cytosolic Ca^2+^ can only increase during repetitive AP’s (Figure 2C). When AP’s are sustained long enough, cytosolic Ca^2+^ remains nearly constant at a steady level (not shown). Ca^2+^ concentration in the cytosol does not exceed 0.4 μM. In this model, repetitive electrical activity always leads to a sustained increase in cytosolic Ca^2+^, which does not correspond to the slow Ca^2+^ oscillations observed experimentally in most α-cells.

The main characteristics of Ca^2+^ dynamics for different values of the K_ATP_ conductance are visible in the bifurcation diagram shown in Figure 2D. The shape of this diagram is most easily understood by looking at the companion bifurcation diagram showing how electrical activity depends on *g*_KATP_ (Figure 2E). Since Ca^2+^ concentration does not feedback on the cell electrical activity in *Model 1*, Ca^2+^ changes can be seen as a simple output of the latter activity. Starting from large values of the K_ATP_ conductance at which cells are hyperpolarized (right part of the diagram), a decrease in this conductance leads to depolarization and electrical firing, as observed in experiments upon the addition of the K_ATP_ channel blocker tolbutamide. A further decline in the K_ATP_ conductance abolishes electrical activity and the cell remains in a constantly depolarized state. This decline in activity allows the model to reproduce the observation that α-cells are electrically inactive in the presence of large concentrations of external glucose without considering paracrine signaling.

The bifurcation analysis allows to uncover the existence of a range of values of *g*_KATP_ for which multiple stable solutions can co-exist. This implies that different steady state solutions can be reached depending on the initial conditions. From 0.2265 to 0.2919 nS (blue region in Figures 2D,E), the system can potentially be in three states: a stable hyperpolarized state, an intermediate stable state (∼ −35 mV) or an electrically active state with large amplitude AP’s. However, the intermediate state, although stable from a physical point of view, is most probably of little physiological significance, as its basin of attraction is very limited. This implies that this state will rarely be reached, and that if reached, fluctuations of internal or external origin would push the system from this intermediate state to the hyperpolarized one or to the limit cycle corresponding to repetitive AP’s. For larger values of *g*_KATP_ (from 0.2919 to 0.3715 nS, green region in Figures 2D,E), the labile, slightly polarized state coexist with the stable hyperpolarized one.

Figure 2C shows that this simple model predicts that when cells are in a stationary regime of repetitive AP’s, cytosolic Ca^2+^ tends to reach a steady state, although very small changes of concentration occur at each AP (∼20 nM). Such rapid and small changes are not expected to be visible experimentally.

In conclusion, slow Ca^2+^ oscillations resulting from intermittent electrical activity as observed in α-cells cannot result from repetitive AP’s controlled by a constant K_ATP_ conductance.

### Randomness in the K_ATP_ Conductance

During electrical activity, the number of simultaneously active K_ATP_ channels is very small, of the order of 10 (Rorsman et al., 2014). Thus, the fluctuations related to internal noise cannot be neglected and the deterministic description of their contribution to membrane current (Eq. 6) must be replaced by a stochastic description. We thus considered the transitions of the channel between an open and a closed state, with a noise on this process as described above (*Model 2* in section “Model of Plasma Membrane Electrical Activity”). Rate constants of channel opening and closing (α and β in Eq. 7) estimated from dwell time distributions are of the order of tenths of ms (Enkvetchakul et al., 2001). Assuming a unitary conductance ($g_{K\,A\,T\,P}^{s}$) of 41 pS (Bokvist et al., 1999; Khan et al., 2001), the total number of K_ATP_ channels in the simulated cell needs to be around 60 to get an average of 5–10 simultaneously open channels during electrical activity, which corresponds to a global K_ATP_ conductance in the range 0.2–0.25 nS. This number fits in the large range of values estimated experimentally (Huang et al., 2011).

**FIGURE 3:**
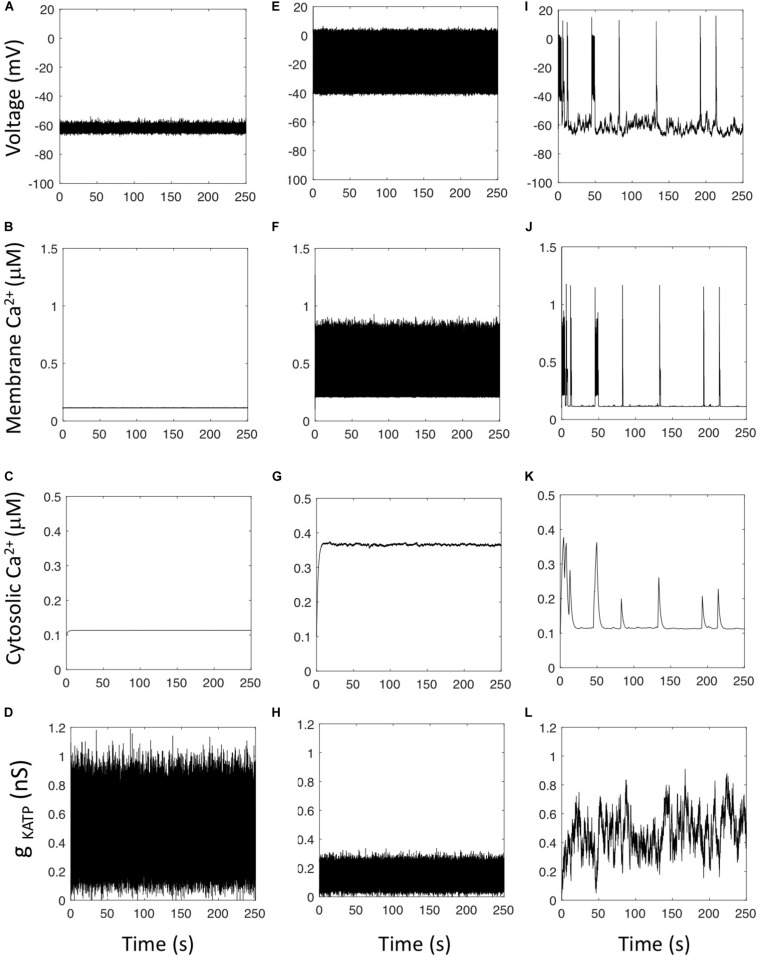
Effect of stochasticity in the K_ATP_ current. Panels show the evolution of the main variables of the model when the conductance of the K_ATP_ channel is described by a Langevin equation (Eqs 7 and 8 with *f*_2_ = 1) to take into account the randomness due to the small number of such channels in α-cells. **(A–D)** Rate constant of opening of the K_ATP_ channel, α = 0.1 ms^–1^, rate constant of closing of the K_ATP_ channel, β = 0.4 ms^–1^; unitary conductance of the K_ATP_ channel$g_{\text{KATP}}^{s}$ = 0.041 nS. The cell is in a quiet, hyperpolarized state despite the large variations of the total cell *g*_KATP_ conductance. **(E–H)** When the unitary conductance of the K_ATP_ channel equals 0.012 nS, the cell is in a depolarized state resulting in a constantly high Ca^2+^ concentration. **(I–L)** When considering slow rates of opening and closing of the K_ATP_ channel, α = 1 10^–4^ ms^–1^ and β = 4 10^–4^ ms^–1^, simulations predict the occurrence of intermittent electrical activity leading to irregular Ca^2+^ spikes in the cytosol. Equations are those corresponding to Model 3 (see section “Outline of the Modeling Approach”) with the default values of parameters listed in the Supplementary Material, except for the indicated values of $g_{\text{KATP}}^{s}$ and _*g_leak*_ = 0.2 nS.

Simulations including noise at the level of K_ATP_ current display AP’s that are variable in amplitude and frequency. When the average value of *g*_KATP_ is in the oscillatory range determined by the bifurcation diagram (Figures 2D,E), the simulated cell is electrically active (average cellular *g*_KATP_ = 0.14 nS in Figures 3E–H), while the cell is in a fluctuating hyperpolarized state when the average value of *g*_KATP_ corresponds to a stable steady state of the bifurcation diagram (average cellular *g*_KATP_ = 0.47 nS in Figures 3A–D). Model simulations never result in Ca^2+^ oscillations whatever the values taken for the unitary conductance of the K_ATP_ channel ($g_{K\,A\,T\,P}^{s}$) and the total number of such channels (*N*_*KATP*_) considered in the simulations. However, we observed that Ca^2+^ oscillations resembling experimental observations could be obtained in the simulations when ascribing to the rate constants of opening and closing of the K_ATP_ channels (parameters α and β) values at least 500 smaller than those reported from experiments (Figures 3I–L). In this case, an intermittent electrical activity generates bursts of AP’s, with each AP leading to a sharp Ca^2+^ increase in sub-membrane Ca^2+^. Sub-membrane Ca^2+^ diffuses in the cytoplasm, and because diffusion is slow, one burst of electrical activity involving multiple AP’s leads to one Ca^2+^ peak in the cytoplasm. Simulations shown in panels I–K display strong resemblance with experimental observations (MacDonald et al., 2007; Quoix et al., 2009; Le Marchand and Piston, 2010, 2012; Zhang et al., 2013; Kellard et al., 2020). The correlation between the cytosolic Ca^2+^ spike and the period of electrical activity has been shown in isolated mouse α-cells (see for example Quoix et al., 2009) and in α-cells from intact islets (Kellard et al., 2020).

From a mechanistic point of view, when the rate constants of opening and closing of the K_ATP_ channels are small, K_ATP_ current variations last long enough to induce a change in the electrical properties of the membrane: when the cell is hyperpolarized, a random decrease in *g*_KATP_ of sufficient duration leads to a cell membrane depolarization that triggers electrical activity and Ca^2+^ entry. Similarly, when the cell membrane is electrically active, a random increase in *g*_KATP_ of sufficient duration leads to a decrease in V that brings the cell in a resting state. With the experimentally reported values of channel opening and closing rates, the random changes in *g*_KATP_ are too fast to induce AP’s. When simulating a stepwise decrease in *g*_KATP_ from 0.47 to 0.2 nS in the absence of noise, the change in *g*_KATP_ must last at least 110 ms to induce electrical activity (not shown). This agrees with the numerical observation that if the fluctuation-driven changes of *g*_KATP_ have a characteristic time of a few ms as measured experimentally (Enkvetchakul et al., 2001), they will not induce AP’s (Figures 2A–C).

Thus, simulations predict that Ca^2+^ oscillations may in principle arise from noise-induced changes in the cell K_ATP_ current. However, the time scale of these changes must be much slower than that of the intrinsic dynamics of the voltage-gated ionic channels generating the AP’s. This does not correspond to the reported rates of opening and closing of the K_ATP_ channels and raises the possibility that some physiological process drives slow changes in the opening of the K_ATP_ channels.

### Ca^2+^-Driven [ATP] Variations Are Responsible for Slow Changes in Electrical Activity

**FIGURE 4:**
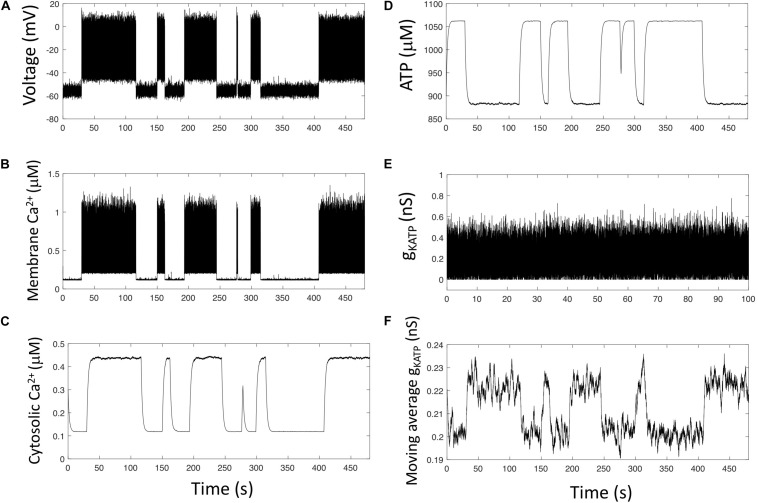
Simulations predict that changes in membrane ATP and stochasticity in the K_ATP_ current generate slow Ca^2+^ oscillations. **(A–E)** Numerical simulations of *Model 3* (see section “Outline of the Modeling Approach”) that describes the opening and closing of the K_ATP_ channel by a Langevin stochastic equation and takes into account the consumption of ATP by PMCA as well as the inhibition of K_ATP_ channels opening by ATP. **(F)** The moving average of *g*_*KATP*_ allows to visualize that, although highly random, *g*_*KATP*_ tends to increase when ATP concentration is low and to decrease when ATP concentration is large. There are thus long-lasting trends in *g*_*KATP*_ changes that can induce robust changes in α-cells electrical activity. Values of *g*_*KATP*_ are averaged on a 200 s period.

K_ATP_ channels are regulated by variations of the intracellular ATP concentration (Bokvist et al., 1999). On the other hand, α-cells show oscillations in Ca^2+^ and ATP submembrane concentrations when observed in constant hypoglycemic conditions (Li et al., 2015). These oscillations are in opposite phase, most probably because Ca^2+^ transport out of the cell is an ATP-consuming process. Here, we investigate the hypothesis that even at constant external glucose, ATP/ADP changes may trigger changes in membrane electrical activity and Ca^2+^ entry that would thus be responsible for the observed, slow cytosolic Ca^2+^ oscillations. To this end, we consider [ATP] as a variable in *Model 3*, as well as its relationship with Ca^2+^ dynamics and its inhibitory effect on K_ATP_ channels conductance. For sake of simplicity, we consider that the conductance of these channels depends on [ATP] via an inhibitory function of the Michaelis-Menten type (Eq. 9). Keeping realistic values for the rates of opening and closing of the K_ATP_ channels (parameters α and β), simulations show highly variable Ca^2+^ oscillations that result from intermittent electrical activity (Figure 4). Each burst of electrical activity triggers a massive entry of Ca^2+^ in the subplasmalemmal compartment (*Ca*_*m*_, Figure 4B), which then invades the cytoplasm (Figure 4C). Because PMCA are fully active, ATP is consumed, and the evolution of its concentration is the mirror image of that of cytosolic Ca^2+^ (Figure 4D). This reduced level of ATP favors a large K_ATP_ current, which in turn reduces electrical activity and Ca^2+^ entry, thus allowing ATP to rise again.

Although fluctuations of the K_ATP_ conductance are very rapid (Figure 4E), *g*_KATP_ is in average larger when [ATP] is low (periods of activity) than when [ATP] is high (quiescent periods) because of the inhibition of the K_ATP_ current by ATP. This is visible when computing the moving average of *g*_KATP_ (Figure 4F). The trend in *g*_KATP_ evolution indeed correlates with that of ATP. Such a trend in the moving average of *g*_KATP_ does not appear when ATP is considered as a constant (*Model 2*, not shown). As a consequence of these trends, large, unlikely perturbations are necessary to switch from an inactive to an active period and *vice-versa*. Thus, the mutual interaction between ATP dynamics and electrical activity that occurs through the Ca^2+^ changes creates trends in the changes in electrical activity allowing for intermittent activity, despite the rapid fluctuations in *g*_KATP_.

**FIGURE 5:**
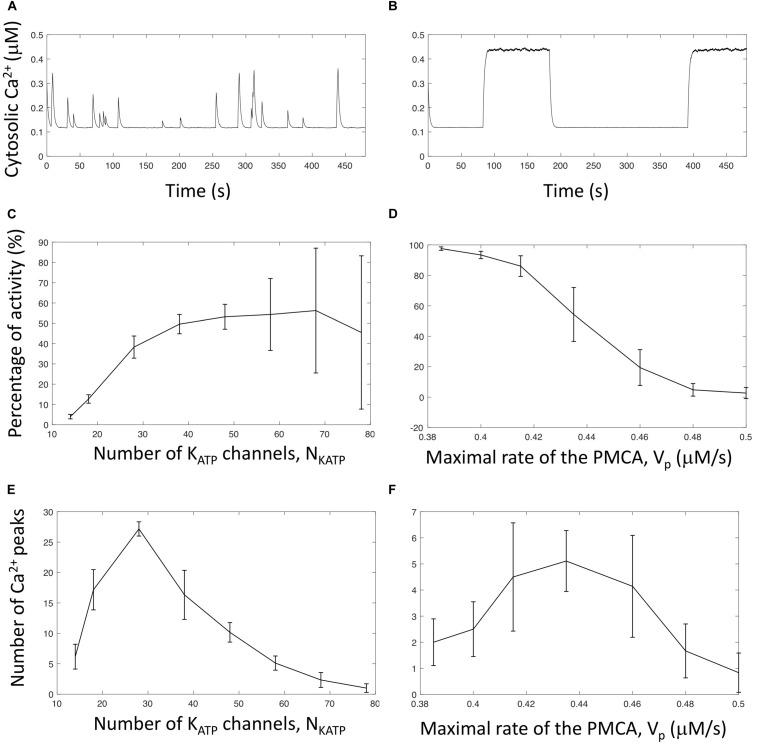
Effect of the number of K_ATP_ channels and rate of PMCA on the pattern of Ca^2+^ oscillations in α-cells. **(A)** Time series of cytosolic Ca^2+^ for *N*_*KATP*_ = 14 and $g_{\text{KATP}}^{\text{s}}$ = 0.170 nS. The maximal global cellular K_ATP_ conductance ($g_{\text{KATP}}^{\text{s}}\cdot N_{\text{KATP}}$) is the same as in Figure 4, but the number of participating channels is smaller. **(B)** Time series of cytosolic Ca^2+^ for *N*_*KATP*_ = 68 and $g_{\text{KATP}}^{\text{s}}$ = 0.03497 nS. The maximal global cellular K_ATP_ conductance ($g_{\text{KATP}}^{\text{s}}\cdot N_{\text{KATP}}$) is the same as in Figure 4, but the number of participating channels is larger. **(C)** Normalized duration of activity as a function of *N*_*KATP*_. **(D)** Normalized duration of activity as a function of the maximal velocity of the PMCA (*V*_*p*_). **(E)** Number of cytosolic Ca^2+^ peaks in a 500 s period as a function of *N*_*KATP*_. **(F)** Number of cytosolic Ca^2+^ peaks in a 500 s period as a function of the maximal velocity of the PMCA (*V*_*p*_). Panels **(C,D)** show the times during which cytosolic Ca^2+^ exceeds 0.3 μM, divided by the total simulation time (500 s). These ratios are expressed in percentages. Six independent simulations have been run for *N*_KATP_ ≤ 58, and 9 for *N*_KATP_ > 58. For panels **(E,F)**, a peak is counted when *Ca*_*c*_ crosses 0.3 μM with a positive slope. Six independent simulations have been run for all values of *V*_*p*_.

The cytosolic Ca^2+^ oscillations shown in Figure 4C have widely different durations, from ∼15 s to ∼2 min. Because of stochasticity, these values are different from one simulation to the other. Given that glucagon secretion is triggered by Ca^2+^ increases above a certain threshold (González-Vélez et al., 2012), it is interesting to investigate what controls the ratio of active *versus* inactive periods. As randomness is required to initiate changes from an active to a quiescent period and *vice-versa*, it can be expected that the number of K_ATP_ channels will play a key role in controlling the number of transitions and thus the activity ratio. To investigate the effect of this factor, we performed simulations with different values of *N*_*KATP*_ and computed the fraction of time during which cells exhibit electrical activity (Figure 5C) as well as the average number of Ca^2+^ spikes during a 500 s simulation (Figure 5E). In the simulations, changes in the values of *N*_*KATP*_ were accompanied by changes in the single channel unitary conductance ($g_{K\,A\,T\,P}^{\text{s}}$) in such a way that the product $N_{\text{KATP}}\cdot g_{\text{KATP}}^{\text{s}}$ remains constant. Given this constraint, the global cell K_ATP_ conductance remains the same for all simulations, which allowed us to only investigate the effect of changes in randomness.

As visible in Figure 5A, when the number of channels is very small (*N*_*KATP*_ = 14), the cell membrane is most of the time hyperpolarized and infrequent, short-duration and low-amplitudes Ca^2+^ spikes occur. If **N*_*KATP*_* is smaller than 10, the cell is always in a resting state, although the average value of the cell K_ATP_ conductance (0.21 nS) is in the oscillatory domain (see Figure 2D). Fluctuations in *g*_*KATP*_ are indeed so important that random depolarizations of the cell membrane are never long enough to initiate an AP. From **N*_*KATP*_* = 10, infrequent Ca^2+^ spikes start to occur. They are very short in duration and amplitude because, once initiated, they are rapidly aborted by a fluctuation that repolarizes the cell. As a consequence, average Ca^2+^ and ATP concentrations are near their resting levels. These concentrations respectively increase and decrease when considering more channels in the simulations, i.e., when randomness is less pronounced. This is due to an increase in both the number of Ca^2+^ peaks (i.e., in the number of bursts of electrical activity, Figure 5E) and the duration of the peaks. Both changes are due to a possibly longer effect of random changes in *g*_*KATP*_ allowing to initiate changes in electrical activity. When **N*_*KATP*_* gets still larger, simulations exhibit a small number of Ca^2+^ spikes of very long duration (Figure 5B). Fluctuations indeed decrease and once in a state, active or resting, the cell tends to remain in this state. In consequence, the number of spikes on a 500 s period becomes smaller (Figure 5E), and the average time of activity larger (Figure 5C). It should be emphasized that for all simulations presented in Figures 5C and E, the average value of *g*_*KATP*_ remains approximatively the same (∼0.21 nS). In the deterministic analysis, this value corresponds to repetitive AP’s (Figure 2D). The different behaviors observed for different values of *N*_*KATP*_ thus exclusively rely on the characteristics of the noise. This computational observation provides an explanation for the widely different profiles of Ca^2+^ oscillations observed in single α-cells that probably express different number of K_ATP_ channels.

In a given cell characterized by a fixed number of K_ATP_ channels, bursts of electrical activity and Ca^2+^ oscillations are sensitive to factors affecting Ca^2+^ dynamics and particularly to the maximal rate of the PMCA, as shown in Figures 5D and F. This velocity indeed affects both Ca^2+^ pumping and ATP hydrolysis. When pumping is slower that in the control situation (Figure 4), Ca^2+^ and ATP concentrations at the membrane remain large, which tends to decrease the number of bursts of activity and increase their duration. Upon an increase in *V*_*p*_, bursts become shorter. Thus, the number of spikes in a given period of time reaches a maximum value. When *Vp* further increases, the decrease in ATP concentration prevents this nucleotide from inducing trends in *g*_*KATP*_ changes and Ca^2+^ spikes finally disappear.

**FIGURE 6:**
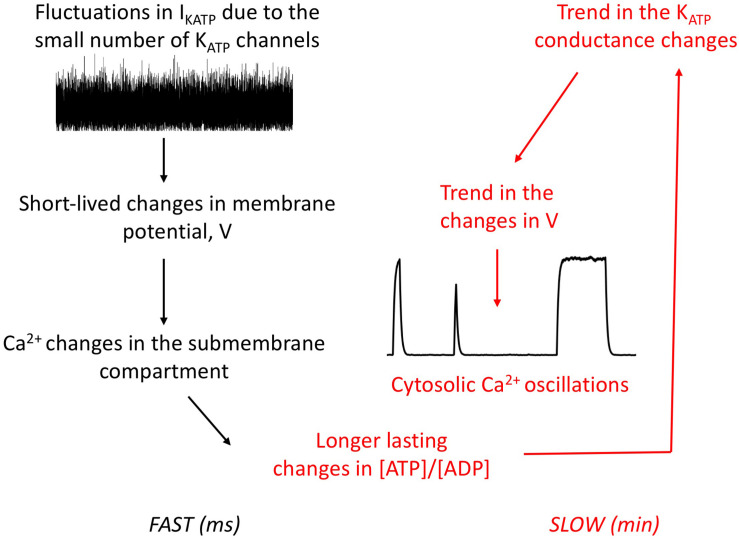
Schematic representation of the interplay between randomness of the K_ATP_ current and ATP changes to generate irregular, slow Ca^2+^ oscillations in α-cells. Rapid fluctuations in the K_ATP_ current due to the small number of channels in the α-cell plasma membrane create fluctuations in membrane voltage that can induce a short-lived change in membrane voltage, which in turn modifies submembrane Ca^2+^ concentration. If the dynamics of ATP is not considered, this noise-induced change in Ca^2+^ would last some milliseconds. If ATP is considered, large fluctuations create a change in ATP concentration of larger duration, because ATP kinetics is much slower than electrical activity. Despite the rapid noise on V that is still present, this will create a trend in the changes in V because of the feedback exerted by ATP on the K_ATP_ current. Thus, periods of quiescence and activity will alternate, which is responsible for cytosolic Ca^2+^ oscillations by diffusion of the membrane Ca^2+^.

Simulations thus predict that the interplay between Ca^2+^ and ATP dynamics that occur through PMCA activity can induce a trend in the noise-initiated changes in K_ATP_ conductance (Figure 6). Rapid fluctuations in the cell K_ATP_ conductance occur because of the small number of such channels involved in the electrical activity of α-cells. The resulting changes in membrane Ca^2+^ induce changes in ATP concentration, because Ca^2+^ and ATP levels in the submembrane space are coupled via the activity of the PMCA. ATP changes in turn feedback on the conductance of the K_ATP_ channels. As the concentration of this nucleotide evolves slowly, these changes are in average maintained on a period of time that is sufficiently long to induce or refrain electrical activity. Thus, the combination of randomness at the level of the K_ATP_ current and of ATP changes can account for the observed long-duration changes in electrical activity and thus for the Ca^2+^ oscillations observed in α-cells.

## Discussion

In low glucose conditions, more than half of α-cells display Ca^2+^ oscillations and these oscillations can persist in high glucose, although much reduced in frequency and amplitude. These Ca^2+^ oscillations are highly variable in shape, duration and interspike interval, not only among different cells but also in the course of time in a given individual cell. Such a randomness strongly suggests the presence of a high level of molecular noise, which generally results from a low number of copies of one or several biochemical species (Gonze et al., 2018). In direct agreement with this theoretical concept, α-cells electrical activity involves a limited number of K_ATP_ channels that play a crucial role in their electrical activity (Rorsman et al., 2014). The aim of this study was to investigate the link between the small numbers of K_ATP_ channels and the existence of irregular, slow cytosolic Ca^2+^ oscillations in α-cells in the rigorous framework of a mathematical model closely based on experimental observations. As Ca^2+^ oscillations are observed not only in α-cells of the intact pancreas but also in isolated cells (Salehi et al., 2006; Quoix et al., 2009; Tuduri et al., 2009; Le Marchand and Piston, 2010), they most probably result from an intrinsic mechanism, modulated by paracrine signaling.

We thus built a model of increasing complexity, on the basis of a previously proposed model of the α-cells electrical activity (Diderichsen and Göpel, 2006). This sequential approach showed that the randomness of the K_ATP_ current does not *per se* allow for Ca^2+^ oscillations. Random changes in this current are indeed too fast to induce any long-term change that could induce intermittent electrical activity. However, when taking into account the triangular relationship between electrical current, Ca^2+^ changes and ATP consumption, the model can account for the electrical and calcium dynamics experimentally observed in α-cells. Moreover, it also accounts for the fact that these dynamics are not stereotypic as quite moderate changes in the number of K_ATP_ channels can induce significant changes in the pattern of Ca^2+^ oscillations. The model predicts that factors that interfere with cell Ca^2+^ changes – such as pumping rates, diffusion coefficients or buffering capacities – may modify the durations during which repetitive AP’s occur. This prediction is relevant for the impaired glucagon response to hypoglycemia and hyperglucagonemia observed in type 2 diabetes.

The simulations predict that the number of simultaneously active K_ATP_ channels during electrical activity is between ∼5 and 10, in agreement with experimental observations (Zhang et al., 2020). This number is much smaller than the total number of channels considered in the simulations (*N*_*KATP*_ = 58), which are all potentially openable. Their intrinsically low open probability and their inhibition by ATP explain why only a small fraction are open simultaneously. Moreover, we expect our value of N_*KATP*_ to be underestimated since we did not take into account effective fatty acid metabolism in low glucose conditions (Briant et al., 2018a) nor the spatio-temporal dynamics of ATP. In any case, that intermittency in electrical activity can be obtained with the model over a relatively large range of values of *N*_*KATP*_, as shown in Figure 5, indicates that the proposed mechanism for Ca^2+^ oscillations in α-cells is robust.

A similar role for stochasticity at the level of the K_ATP_ current has been put forward to account for the irregularity of the neuronal firing pattern in hippocampal CA3 neurons (Huang et al., 2007). However, in this case, the changes in K_ATP_ current are due to rapid, random fluctuations in the concentration of ATP, which results in an irregular frequency of AP’s and not in intermittent electrical activity. In the more general context of networks dynamics, it is known that fluctuations can propagate along the different nodes of the network with a rate of decay or enhancement that depends on the network’s structure (Maslov et al., 2007; Zhang et al., 2012).

Within this study, we did not investigate the behavior of the model when changing external glucose or considering paracrine signaling, which are left for further study. These limitations are due to the fact that the model used for electrical activity (Diderichsen and Göpel, 2006) does not reproduce one of the key characteristics of α-cells when raising external glucose, i.e., the reduction in the amplitude of the AP’s (see Ashcroft and Rorsman, 2013 for example). This reduction leads to less activation of the Ca^2+^ channels linked to glucagon secretion. In Figure 2, this reduction was simulated by an artificial change in the leak conductance when changing the conductance of the K_ATP_ channels to simulate the changes in external glucose. However, given the reliability of the Diderichsen and Göpel’s model and the many unknowns in the field of α-cells dynamics, we considered this unchanged model as a safe starting point to explore the impact of a stochastic K_ATP_ current and of changes in ATP concentration. However, we acknowledge that values of parameters for the ionic currents obtained with better methods of α-cell identification could improve the agreement between the simulations and the experiments (Briant et al., 2017; Montefusco et al., 2020).

The hydrolysis of ATP that parallels Ca^2+^ extrusion out of the cell plays a key role in the behavior predicted by the model. However, ATP synthesis is also sensitive to intracellular Ca^2+^ changes because mitochondrial metabolism is Ca^2+^-sensitive (Wacquier et al., 2019). The ATP/ADP ratio is also controlled by glycolysis and fatty acid metabolism (Briant et al., 2018a). Further studies are required to address these interrelationships in a more accurate way (Olivos-Santes et al., 2019), although the qualitative results obtained with the relatively simple model used in this study should be robust towards these numerous possible model refinements.

As another limitation, we only considered noise at the level of the K_ATP_ channel, while all molecular processes, and especially Ca^2+^ dynamics, are subjected to noise. This simplification was based on the experimental observation of an unusually small K_ATP_ conductance in α-cells, in particular in comparison with pancreatic β-cells that are known to be equipped with the same channels but still display very regular voltage and calcium patterns. However, the details of our conclusions may be affected by adopting a full stochastic description of the cell dynamics. In particular, it may lead to less stereotypic cytosolic Ca^2+^ changes, as reported in the experiments.

An interesting perspective would be to couple the model presented in this study to our previously proposed model that relates glucagon secretion to cytosolic Ca^2+^ changes (González-Vélez et al., 2012). The latter model is able to predict glucagon secretion using as an input experimentally obtained Ca^2+^ traces. It was found that glucagon secretion does not correlate with the frequency of Ca^2+^ oscillations. This investigation could be pursued by using Ca^2+^ time series obtained by simulations of the present model instead of experimental data. This could lead to identify the key elements of electrical and/or calcium activities controlling glucagon secretion.

## Data Availability Statement

The original contributions presented in the study are included in the article/Supplementary Material, further inquiries can be directed to the corresponding author.

## Author Contributions

VG-V, AP, and GD contributed to the conceptualization, design of the study, development, simulation, and analysis of the models. GD and VG-V contributed to the preparation of the manuscript. All authors contributed to the article and approved the final version.

## Conflict of Interest

The authors declare that the research was conducted in the absence of any commercial or financial relationships that could be construed as a potential conflict of interest.
