# Supplementary material for: Calcium Oscillations in Pancreatic α-cells Rely on Noise and ATP-Driven Changes in Membrane Electrical Activity
Source: Front Physiol. 2020 Nov 17;11:602844. doi: 10.3389/fphys.2020.602844 (PMC7705205; doi:10.3389/fphys.2020.602844)
Supplement: Supplementary file 1 [file Data_Sheet_1.PDF]

Supplementary Material

1 Supplementary Data

Equations of the model of Diderichsen and Göpel (2006)

We use the model proposed by Diderichsen and Göpel (2006) to describe electrical activity of α-cells. We also consider a N-type Ca^{2+} current, described as in Csercsik et al. (2010). A detailed description of the model can be found in the original work. Here below, we list the equations of the model.

The evolution of membrane voltage is given by:

$$\frac{dV}{dt} = -\left(I_{CaT} + I_{CaL} + I_{CaN} + I_{Na} + I_{KA} + I_{KDR} + I_{KATP} + I_{leak}\right)/C_m \qquad \text{(S1)}$$

Each current is described by the Hodgkin-Huxley formalism, i.e.

$$I_{Ca,T} = \bar{g}_{Ca,T} m_{Ca,T}^3 h_{Ca,T} (V - V_{Ca}) \qquad \text{(S2)}$$

$$I_{Ca,L} = \bar{g}_{Ca,L} m_{Ca,L}^2 h_{Ca,L} (V - V_{Ca}) \qquad \text{(S3)}$$

$$I_{Ca,N} = \bar{g}_{Ca,N} m_{Ca,N}^2 h_{Ca,N} (V - V_{Ca}) \qquad \text{(S4)}$$

$$I_{Na} = \bar{g}_{Na} m_{Na,V}^3 h_{Na,V} (V - V_{Na}) \qquad \text{(S5)}$$

$$I_{K,A} = \bar{g}_{K,A} m_{K,A}^3 h_{K,A} (V - V_K) \qquad \text{(S6)}$$

$$I_{K,DR} = \bar{g}_{K,DR} m_{K,DR}^4 (V - V_K) \qquad \text{(S7)}$$

$$I_{K,A} = \bar{g}_{K,A} m_{K,A}^3 h_{K,A} (V - V_K) \qquad \text{(S8)}$$

The conductance of the K_{ATP} and leak current are assumed to remain constant and thus,

$$I_{K,ATP} = \bar{g}_{K,ATP} (V - V_K) \qquad \text{(S9)}$$

$$I_{leak} = \bar{g}_{leak} (V - V_{leak}) \qquad \text{(S10)}$$

The gating functions *m* and *h* appearing in (S2)-(S8) have been obtained by fitting experimentally recorded currents to simulated currents. The details of these functions and their parameter values can be found in the Appendix of Diderichsen and Göpel (2006). Values of the other parameters appearing in equations (S2)-(S8) are listed in Table 1.

Estimation of the linear diffusion coefficient

The 1-dimensional equation for diffusion,

$$\frac{\partial Cam}{\partial t} = -D\frac{\partial^2 Cam}{\partial x^2} \quad \text{(S11)}$$

can be approximated by

$$\frac{\partial Cam}{\partial t} = -\frac{D}{h^2}(Cam - Cac) = -\gamma(Cam - Cac) \quad \text{(S12)}$$

where h stands for the distance between the sub-membrane and the cytosolic compartments.

D=13 $\mu m^2/s$ (Allbritton *et al.*, 1992). The average diameter of an α-cell is 8 μm and the diameter of the nucleus is about 70% of that of the cytosol (Zimny and Blackare, 1975), one can estimate γ as

$$\gamma \approx \frac{13}{(4-2.8)^2} \cdot 0.001 = 0.0090278\, ms^{-1} \quad \text{(S13)}$$

In the simulations, we have thus used γ =0.01 ms^{-1}.

References

Allbritton, N., Meyer, T., and Stryer, L. (1992). Range of messenger action of calcium ion and inositol 1,4,5-trisphosphate. Science 258, 1812-1815.

Diderichsen, P., and Göpel, S. (2006). Modelling the electrical activity of pancreatic α-cells based on experimental data from intact mouse islets. J. Biol. Phys. 32, 209-229. doi: 10.1007/s10867-006-9013-0

Zimny, M., and Blackard, W. (1975). The surface structure of isolated pancreatic islet cells. Cell Tissue Res. 164, 467-471.

2 Supplementary Tables

Table 1. Values of the parameters of the model of Diderichsen and Göpel (2006) for α-cell electrical activity.

Parameter	Value	Unit
C_m	5	pF
V_{Na}	110	mV
V_K	-80	mV
V_{Ca}	60	mV
V_{leak}	-20	mV
$\bar{g}_{Ca,L}$	1	nS
$\bar{g}_{Ca,T}$	1	nS
$\bar{g}_{Ca,N}$	1	nS
$\bar{g}_{K,A}$	20	nS
$\bar{g}_{K,DR}$	4.9	nS
$\bar{g}_{Na}$	10	nS
$\bar{g}_{leak}$	0.13	nS

Table2. Default values of the parameters characterizing Ca^{2+} and ATP dynamics used in the simulations

Parameter	Value	Unit
f_R	0.1	-
F	96587	$Cmol^{-1}$
Vol	623.61	μm^3
V_b	0.04	μMms^{-1}

$\boldsymbol{V_p}$	0.435	μMms^{-1}
$\boldsymbol{K_m}$	0.35	μM
$\boldsymbol{K_e}$	0.05	μM
$\boldsymbol{V_{gly}}$	1.2	μMms^{-1}
$\boldsymbol{Glu}$	1	mM
$\boldsymbol{K_{glu}}$	1	mM
$\boldsymbol{k}$	5.4 10^{-4}	μMms^{-1}
$\boldsymbol{\alpha}$	0.1	ms^{-1}
$\boldsymbol{\beta}$	0.4	ms^{-1}
$\boldsymbol{N_{KATP}}$	58	-
$\boldsymbol{K_{inh}}$	620	μM
$\boldsymbol{g^s_{KATP}}$	0.041	nS
